# Measuring Efficiency of Semi-automated Brain Tumor Segmentation by Simulating User Interaction

**DOI:** 10.3389/fncom.2020.00032

**Published:** 2020-04-16

**Authors:** David Gering, Aikaterini Kotrotsou, Brett Young-Moxon, Neal Miller, Aaron Avery, Lisa Kohli, Haley Knapp, Jeffrey Hoffman, Roger Chylla, Linda Peitzman, Thomas R. Mackie

**Affiliations:** HealthMyne Inc., Madison, WI, United States

**Keywords:** brain MRI, tumor, segmentation, glioma, deep learning, efficiency

## Abstract

Traditionally, radiologists have crudely quantified tumor extent by measuring the longest and shortest dimension by dragging a cursor between opposite boundary points across a single image rather than full segmentation of the volumetric extent. For algorithmic-based volumetric segmentation, the degree of radiologist experiential involvement varies from confirming a fully automated segmentation, to making a single drag on an image to initiate semi-automated segmentation, to making multiple drags and clicks on multiple images during interactive segmentation. An experiment was designed to test an algorithm that allows various levels of interaction. Given the ground-truth of the BraTS training data, which delimits the brain tumors of 285 patients on multi-spectral MR, a computer simulation mimicked the process that a radiologist would follow to perform segmentation with real-time interaction. Clicks and drags were placed only where needed in response to the deviation between real-time segmentation results and assumed radiologist's goal, as provided by the ground-truth. Results of accuracy for various levels of interaction are presented along with estimated elapsed time, in order to measure efficiency. Average total elapsed time, including loading the study through confirming 3D contours, was 46 s.

## Introduction

Malignant brain tumors often have unfavorable prognoses such as time to progression and overall survival, and also have direct impact on motor and/or cognitive function and poor quality of life (Omuro and DeAngelis, 2013). In recent decades, imaging has played a key role throughout the entire treatment paradigm of cancer patients ranging from diagnosis and presurgical planning to treatment response assessment. Additionally, multimodal MRI protocols allow for non-invasive interrogation of tumor heterogeneity and identification of phenotypic sub-regions i.e., peritumoral edema/invasion, enhancing active tumor core and necrotic regions which reflect tumor biological properties including tumor cellularity, vascularity, and blood-brain barrier integrity.

However, despite the exponential enhancement in imaging sequences, hardware and software, we have barely begun to tap the potential of non-invasive imaging to characterize the phenotype of tumors. To date, radiologic assessments are qualitative including tumor detection and image-based tumor staging or semi-quantitative using freehand uni-dimensional and bi-dimensional measurements of the tumor. In fact, all current imaging assessment criteria [such as Response Evaluation Criteria in Solid Tumors (RECIST), Response Assessment in Neuro-Oncology (RANO), immune related RECIST (irRECIST), and immune related response criteria (irRC)] used to evaluate tumor response in the clinical setting or in clinical trials rely on these freehand measurements to evaluate tumor size (Sorensen et al., 2008; Eisenhauer et al., 2009; Wolchok et al., 2009; Wen et al., 2010).

Accurate assessment of tumor volume is important for clinical management and particularly for monitoring treatment response and development of new therapies and trials. Despite the well-known advantages of whole tumor volumetric assessment, as recognized by the RANO Working Group, currently it is only performed for research purposes as manual outlining can be time-consuming, and it is susceptible to inherent intra-observer and inter-observer variability (Wen et al., 2010). Research efforts have focused on the development of computer-aided techniques for tumor segmentation. Computer-aided tumor segmentation techniques can be grouped in two major categories based on the radiologist/user interaction with the tool; (1) fully automated techniques that require no, or negligible user input, and (2) semi-automated techniques that require some localization or initialization from the user; then the algorithm provides the majority of segmentation optimization. Semi-automated techniques outperform automatic approaches, resulting in sufficiently accurate and robust results (Zhao and Xie, 2013). However, semi-automated techniques do not scale well to large number of labeled datasets, since developing and validating interactive algorithms becomes laborious as the datasets grow. Consequently, there is an unmet need for an approach to simulate user interaction that will allow for efficient and cost-effective evaluation of semi-automated techniques throughout the development and validation stages.

In a recent publication we presented Semi-Automated Map-BAsed Segmentation (SAMBAS), which allows for real-time feedback by an expert radiologist (Gering et al., 2018). In short, the user initializes the segmentation process by drawing a long axis; during the long axis drawing, the 2D segmentation updates in real-time for interactive feedback. In cases of suboptimal 2D segmentation the user can refine the result by drawing a short axis. Further optimization can be performed on the other two planes prior to 3D segmentation initialization. This interactive system outperformed the Deep Learning (DL) approach alone; as demonstrated in our publication, using the Multimodal Brain Segmentation Competition (BraTS) 2018 validation data the interactive system resulted in an improved Dice similarity coefficient over DL alone and the lowest Hausdorff-95% distance on the BraTS leaderboard (Menze et al., 2015; Bakas et al., 2017a, 2018; Gering et al., 2018).

However, it is still unknown how real-time experiential input affects Dice coefficient and Hausdorff-95% distance. Therefore, in this study, we designed an experiment to simulate the level of user interaction. Specifically, we used the 2018 BraTS training data as the ground-truth and a computer simulation mimicked the process that a radiologist would follow to perform segmentation with real-time interaction (Bakas et al., 2018). Clicks and drags were placed only where needed in response to the deviation between real-time segmentation results and assumed radiologist's goal, as provided by the ground-truth. Results of accuracy for various levels of interaction are presented along with estimated elapsed time, in order to measure efficiency.

## Materials and Methods

Rapid Precise Metrics™ (RPM) implements an interactive algorithm as a probabilistic framework with efficient user interaction and control in the HealthMyne® Platform (HealthMyne, Madison, WI). Additional details on how RPM seamlessly merges DL with user interaction can be found on Gering et al. (2018). For the purposes of this work, we removed the DL component because access was needed to the same ground-truth data on which the DL would have trained. Indications by a skilled radiologist are another aspect of RPM missing from this experiment. Consequently, the absolute values of accuracy reported do not fully reflect clinical performance of RPM, however, relative accuracy and timing measurements should be representative.

The organization of the manuscript is as follows: the system for interactive Multi-Plane Reformat (MPR) segmentation will be described first, followed by the method for simulating a user's interaction with the system. Finally, the method for performing timed tests will be presented.

### Interactive Multi-Plane Reformat (MPR) Segmentation

**Figure 1:**
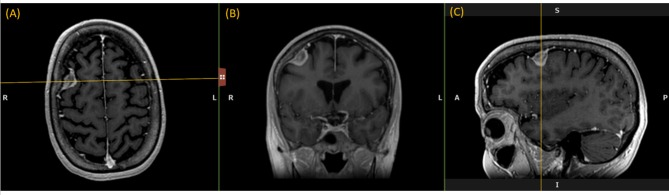
Multi-Plane Reformat: an example of Multi-Plane Reformat (MPR) slices of the 3D volume corresponding to axial, coronal and sagittal planes. The yellow lines denote the position of the coronal plane **(B)**, with respect to the axial plane **(A)** and the sagittal plane **(C)**.

Like a digital simulation of a traditional light box on which radiologists formerly viewed film, the 3D volume is visualized by displaying 2D planes sequentially. A MPR refers to reformatting more than one plane, such that a trio of planes is displayed side-by-side corresponding to axial, coronal, and sagittal orientations (Figure 1).

**Figure 2:**
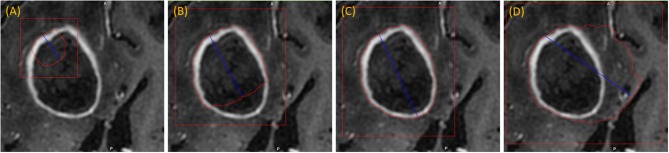
Interactive segmentation: several instances during interactive segmentation **(A–D)** depicting the segmentation contour (red) updating in real-time as the user drags the endpoint of the long axis (blue). In **(C)** a correct segmentation of core tumor is displayed, while **(D)** displays response to overdrawing.

The user initializes the segmentation process by drawing a long axis on one plane of the MPR. As the user draws the long axis, a 2D segmentation updates in real-time for interactive feedback. The feedback has proven to be very helpful for the user to know precisely where to place the endpoint of the axis (Gering et al., 2018). Upon release of the mouse, 2D segmentation occurs immediately on the other MPR planes. Figure 2 shows the interactive feedback.

When the 2D contour is unsatisfactory, additional drags may be drawn to complement the long axis. Furthermore, single clicks may be used to “drop” points along the structure boundary. Another available editing operation is a “ball tool” for drawing with a digital brush. A correct 2D segmentation is important since probability distributions are learned from the 2D segmentation and employed in segmenting the other MPR planes.

When the contours on other MPR planes are unsatisfactory, then the user can further refine the segmentation by either drawing long axes on these planes, or by editing the segmentation masks with the ball tool. This is especially useful for irregularly shaped lesions or lesions oriented obliquely to the anatomical axes. Once initial segmentation is satisfactory, the user can initiate 3D segmentation by a single click.

3D segmentation occurs quickly (approximate time = 1–2 s), and the user may inspect the resulting contours by scrolling through slices on any MPR plane. If unsatisfied, the user has two options, either delete the lesion segmentation and re-draw a better long axis, or alternatively edit the 3D segmentation using a 3D sphere tool. When satisfied, the user clicks a button to confirm the 3D contours.

### Simulation System

The simulation system automatically draws a long axis on each tumor. Depending on the accuracy of the resulting segmentation, more drags or clicks are added as needed. The process by which these are drawn aims to mimic a human user's actions, as described below.

#### Data Preparation

Multi-institutional, routine clinically-acquired, pre-operative, multispectral MR scans were provided by the 2018 BraTS challenge (Bakas et al., 2017b,c, 2018). The data have been preprocessed to be co-registered to the same anatomical template, interpolated to the same resolution (1 mm^3^), and skull-stripped.

For the purposes of this work, we used only the post-contrast T1-weighted MR scans. While BraTS provided labels for three phenotypes: whole tumor, core tumor, and active tumor, we combined the ground-truth masks for “core” and “active” to form “core tumor,” and used this one tumor component exclusively in this experiment, since this is representative of the gross tumor extent assessed by a radiologist in the clinical setting.

#### Enumerating Tumors

**Figure 3:**
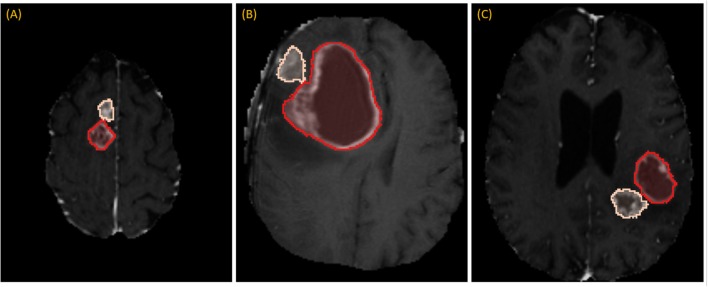
Separating adjoining tumors: three examples of adjoining tumors **(A–C)** which were manually separated to form distinct tumors, shown here in tan and red.

While RPM is designed to segment individual lesions, BraTS ground-truth presents a unified mask without separating individual lesions. Therefore, we manually drew blank (zero-valued) lines to separate adjoining lesions. After this one manual step, lesions could be enumerated automatically by running 3D connected-component analysis (CCA) to identify each distinct “island” of the ground-truth mask, as illustrated in Figure 3. Given the 285 patients, 232 had 1 lesion, 33 had 2 lesions, and 20 had 3 or more, with the maximum being 5.

#### Simulating the Drawing of a Long Axis

The first step toward drawing a long axis is selecting the axial slice on which to draw. Our aim was to replicate the approach of an expert radiologist briefly scrolling through the slices to eyeball the one on which the tumor appears the largest. In the first step, for each enumerated tumor, the range of slices containing ground-truth was found, and the subset of slices in the central third was considered. Given this subset, the slice with the largest area of ground-truth was chosen.

In order to simulate the type of long axis that a user might draw, we employed four different methods: (i) identification of the true longest axis, (ii) selection of an axis that is located more medially than the true longest axis, (iii) search for an axis that includes pixels that statistically typify the tumor, and (iv) sweeping a short distance to search for optimal results.

**Figure 4:**
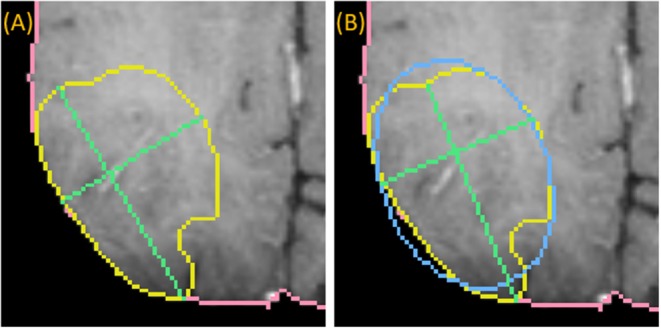
Medially placed longest axis: a demonstration of a more medially placed longest axis obtained by fitting an ellipse (blue) to the ground-truth (yellow) and finding the long and short axes (green) parallel to the major and minor axes of the ellipse. The true longest axis is drawn in **(A)**, while the more medially positioned axis is drawn in **(B)**.

To draw the “medial” long axis, an ellipse is fit by Principle Component Analysis (PCA) to the 2D segmentation (Duda et al., 2012). The long axis with the same orientation as the major axis of the ellipse is selected, as shown in Figure 4. This method is driven by the fact that RPM tends to perform better on centrally located drags where symmetry can be exploited.

Since RPM samples statistics under the long axis, it's important to consider that aspect in addition to length and centrality. Therefore, the third method searches the set of *N*^2^ possible axes drawn between a set of *N* points spaced near each endpoint of the medial axis. Based on the ellipse, these points are spaced by a few degrees, and lie on the boundary of the ground-truth. A score is computed for each axis, and the axis with the best score is selected. Equation (1) describes the score as a weighted combination of properties of the long axis, namely length, centrality, and relative entropy, also referred to as Kullback-Leibler divergence (*D*_*KL*_) (Cover and Thomas, 2012).

(1) $$\begin{array}{l} Score=\alpha *\left(1-D_{KL}\right)+\beta *Length+\gamma *Centrality \end{array}$$

where α, β and γ are scalar parameters. Since the Kullback-Leibler divergence is a measure of how one probability distribution is different from another, it is an appropriate metric for evaluating how well the pixels along the long axis relate to the pixels of the entire 2D structure. Equation (2) expresses this relationship where the probability distribution, *Q*, of pixels sampled under the long axis is estimated by Parzen window density estimation, and the probability distribution, *P*, is estimated similarly from pixels sampled under the ground-truth mask (Duda et al., 2012).

(2) $$\begin{array}{l} D_{KL}\left(P\|Q\right)=\;-\underset{x\in X}{\sum }P\left(x\right)log\left(\frac{Q\left(x\right)}{P\left(x\right)}\right) \end{array}$$

The *Length* and *Centrality* in Equation (1) are terms with a range [0, 1] and are computed as follows:

(3) $$\begin{array}{l} Length=\frac{length\;of\;axis}{length\;of\;true\;longest\;axis} \end{array}$$

(4) $$\begin{array}{l} Centrality=0.1+0.9*C/\left(2r\right) \end{array}$$

Where *C* encodes the distance from center of ellipse obtained for Equation (5):

(5) $$\begin{array}{l} C=|i-r|+|j-r| \end{array}$$

**Figure 5:**
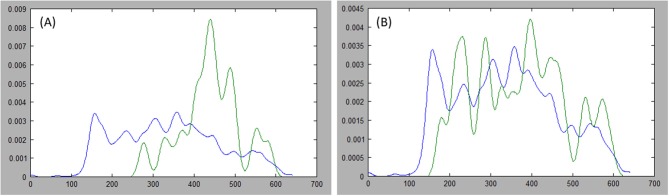
Comparing distributions: estimations of the probability distribution function (PDF) for tumor ground truth and pixels under the long axis are plotted. A high discrepancy is plotted in **(A)** resulting in large Kullback-Leibler (KL) divergence, while the PDFs are more similar in **(B)** resulting in small KL divergence.

Where indices, *i* and *j*, index the sets of *N* points on each side of the axis, and *r* represents the index, *N*/2, of the middle point in a set. Furthermore, we favor axes that cross the center by halving the expression above when *i* and *j* both lie on the same side of *r*. Figure 5 presents examples of probability distribution functions for a long axis that is representative of the tumor, and another axis that is divergent.

**Figure 6:**
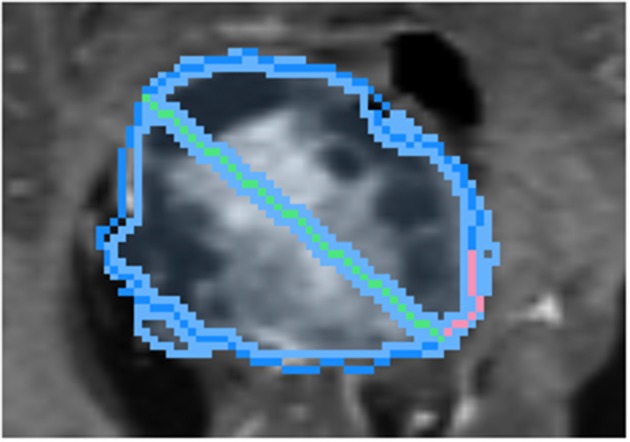
Demonstration of sweeping approach for long axis identification: while the leftmost endpoint of the long axis (green) is held fixed, the rightmost endpoint is swept along a short path (pink) along the boundary of ground-truth (dark blue). At each position, the similarity between the segmentation (light blue) and ground-truth is measured.

To draw the “swept” long axis, the first endpoint of the long axis is held fixed while the second endpoint is dragged along the boundary of the structure, by a short distance in each direction, as shown in Figure 6. At each position during the sweep, the interactive 2D segmentation is performed, and the position with the best comparison with ground-truth is selected (by DSC defined below).

#### Simulating the Drawing of a Short Axis

While the RPM algorithm allows multiple drags of any orientation, we simplified this experiment by drawing only the “short axis,” which is defined as the longest axis that lies perpendicular to the long axis.

#### Simulating Dropped Points Along Structure Boundary

Given a segmentation based on the long and short axes, the contour point of greatest disagreement with the ground-truth is identified. Subsequently, an editing operation is performed by “dropping” a point on the structure boundary as indicated by the ground-truth. As described earlier, these drops serve as inputs into RPM's algorithm that are quicker to draw than a line with two endpoints.

**Figure 7:**
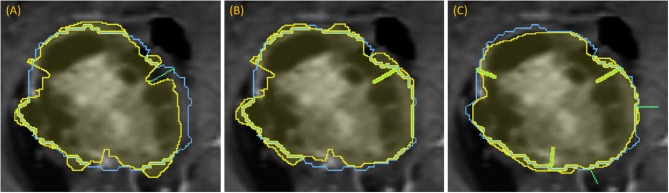
Demonstration of dropping points: segmentation (yellow) relative to the ground-truth outline (blue) for a progression of dropping points to segment a complex lesion: **(A)** green line denotes where a dropped point is most needed, **(B)** updated segmentation after dropping that point, **(C)** updated segmentation after dropping five points, each indicated with a green line.

Following the first dropped point, segmentation is recomputed and the next contour point of greatest disagreement is identified, if any, as there may be no remaining significant discrepancies. New points cannot be placed too closely to earlier points. In this manner, more points can be “dropped” in succession, triggering new segmentations with each dropped point (Figure 7).

#### Simulating Drawing on MPR

The center of the long axis is used to determine the center of the reformatted sagittal and coronal planes that comprise the 3-plane MPR. Long and short axes were drawn in similar manner on all planes. The additional axes precipitate MPR segmentation.

### Comparison of Volumes

While each tumor is segmented individually, the “ground-truth” is provided per patient rather than per lesion. Consequently, we took the union of the segmentations of all lesions for each patient, and compared these aggregates with the “ground-truth” for agreement. The Dice similarity coefficient (DSC) was used to measure the similarity between two sets of segmentations and was calculated using the Equation (3):

(6) $$\begin{array}{l} DSC=\;\frac{2\left(A\cap B\right)}{\left(A+B\right)} \end{array}$$

where A represents the semi-automated segmentation and B represents the “ground-truth” (Allozi et al., 2010). Scores were computed by uploading segmentations to the CBICA Image Processing Portal.

### Timing Tests

To estimate an average elapsed time, the entire population was partitioned into three categories so that a weighted average could be computed where the weights are determined based on the size of each category. From each category, 10 cases were randomly sampled (by Python script) to be segmented by a human user with the interactive system; the user has extensive background in the design and implementation of clinical software solutions, though no specific radiology training. The total elapsed time measured included all of the tasks listed in Table 1, which span from beginning to load the study to confirming 3D contours.

**Table 1:** Timed tasks.

Load study
Scroll to lesion
Segment by dragging long axis
Optionally open MPR view for additional drags/clicks
Perform 3D segmentation
Scroll to inspect 3D contours
Confirm 3D contours

The three categories were the following: (i) cases that segmented well with drawing an axial long axis (DSC > 0.883; *n* = 109); (ii) cases that required drawing long axis on all MPR planes (*n* = 123); (iii) cases that required additional edits (*n* = 53). Prior to the random selection, the categories were whittled down in size by more restrictive criteria in order to form subsets of patients for which the interaction was more meaningful. These subsets were cases that segmented extremely well (DSC >= 0.93) with a single drag (*n* = 39), cases whose score increased by at least 0.05 to achieve a total score of at least 0.85 given a long axis drag on all MPR planes (*n* = 25), and cases whose score increased further by at least 0.05 to achieve a total score of at least 0.85 given additional edits on MPR (*n* = 41).

To evaluate the realistic nature of simulations against real user interaction, DSC score from manual segmentation was compared against DSC obtained from simulations. First, the fitness of DSC scores to normal distribution was determined by Kolmogorov-Smirnov test, and then the scores were compared using *t*-test or Mann-Whitney test accordingly. A *p*-value of < 0.05 was considered statistically significant. Finally, for completeness we calculated the DSC score between the segmentation obtained from real user interaction and from simulation.

## Results

Of the 285 patients, 232 had a solitary lesion, and 53 had more than one lesion resulting in a total of 365 brain lesions available for segmentation.

### Long Axis Simulation

We simulated four different strategies for drawing the long axis: (i) obtaining the true longest axis, (ii) assigning the long axis more medially than the true longest axis, (iii) searching for an axis that statistically typifies the tumor, and (iv) sweeping a short distance to search for optimal results. Using the aforementioned strategies as initialization step, the 3D segmentations were compared with the “ground-truth.” Table 2 summarizes the results for DSC between the four strategies, with swept being noticeably superior (DSC = 0.821).

**Table 2:** Comparison of various strategies for simulating the drawing of the long axis.

**Long axis style**	**3D dice**
**True longest**	
Mean	0.798
St. Deviation	0.130
Range	[0.315–0.972]
**Favoring medial position**	
Mean	0.812
St. Deviation	0.122
Range	[0.232–0.963]
**Searching for statistics**	
Mean	0.807
St. Deviation	0.130
Range	[0.232–0.963]
**Sweeping one endpoint**	
Mean	0.821
St. Deviation	0.120
Range	[0.305–0.972]

### Simulating User Interaction

We simulated a varied degree of user interaction from drawing only one axis to editing 3D segmentation to perfection. Additionally, simulations allowed for drawing on the axial plane only, or on all 3 MPR planes: axial, coronal, and sagittal. Table 3 summarizes the results of 3D segmentations with varying degrees of user interaction. Our results indicate that drawing long axes on MPR planes compared to drawing only one long axis on the axial plane resulted in significantly (by at least 0.05) improved DSC scores in 76 patients out of a total of 285. Similarly, drawing short axes and dropping points on MPR planes resulted in significantly higher DSC scores in 88 patients, while in 14 patients the DSC score worsened significantly. Finally, editing segmentation outcome to perfection on MPR planes significantly improved the DSC score in 123 patients while significantly worsening only 2.

**Table 3:** Varying levels of interaction.

**User input**	**Axial plane only**	**All 3 MPR planes**
**Long axis only**		
Mean	0.821	0.851
St. Deviation	0.120	0.079
Range	[0.305–0.972]	[0.517–0.965]
**Long and short axes**		
Mean	0.823	0.858
St. Deviation	0.110	0.068
Range	[0.385–0.973]	[0.512–0.965]
**Long and short axes and few dropped points**		
Mean	0.834	0.864
St. Deviation	0.103	0.063
Range	[0.309–0.973]	[0.557–0.965]
**Edited to perfection**		
Mean	0.839	0.890
St. Deviation	0.105	0.050
Range	[0.431–0.970]	[0.681–0.970]

Table 4 presents results from a few intermediate stages of the algorithm. For responsive interaction, RPM segments first in 2D, corresponding to the first row of Table 4, and then it initializes the 3D segmentation by segmenting on a 3-plane “scout” reformat, corresponding to the second row of the Table 4, and then it finally segments in 3D. There is a column for each level of user interaction.

**Table 4:** Progression through processing stages.

**Stage**	**True long**	**Sweep long**	**Long and short**	**Long, short and drops**	**Axial perfect**	**MPR perfect**
2D	0.886	0.916	0.919	0.947	0.972	0.968
3-plane Scout	0.845	0.859	0.866	0.878	0.886	0.960
3D	0.798	0.821	0.823	0.834	0.839	0.890

### Timing Tests

The average elapsed time for each patient in the entire population was estimated to be 46.2 s; this was a weighted average computed over three categories whose boundaries were described in section Timing Tests. The number of cases in the first category (drawing an axial long axis) is the total number of cases whose scores were above 0.883, which was 109. The number of cases in the second category (drawing long axes on all MPR planes) was the number of the remaining patients whose scores were at least 0.796 given MPR drags, which is 123. Finally, 53 cases comprised the third category (additional edits required).

Table 5 summarizes the results of measuring elapsed time for a user to segment a batch of 10 cases from each of the three categories of interaction. Regarding the first category where the long axis is drawn only on the axial plane, the average time the segmenter reported was 30.38 s, ranging from 23.65 to 44.92 s. One of the 10 cases was large and highly heterogeneous, and therefore the user had more slices to sort through to determine a good place to drag; it should be noted that the initial drag for this case was deleted and redrawn from a better angle. Similarly, drawing the long axis on all three MPR planes resulted in an average time of 52.0 s (range: 31.75–86.61 s). In the final category, dragging lines and dropping points, as well as drawing using the ball tool, as needed on MPR, resulted in an average time of 65.31 s (range: 46.73–107.75 s).

**Table 5:** Timing measurements.

**User input**	**Mean elapsed time (seconds)**	**DSC (user vs. ground-truth)**	**DSC (simulation vs. ground-truth)**	***p*-value**	**DSC (user vs. simulation)**
**Long axis only**					
Mean	30.38	0.934	0.943	0.1041	0.951
St. Deviation	6.41	0.013	0.008		0.021
Range	[23.65–44.92]	[0.911–0.952]	[0.933–0.954]		[0.918–0.985]
**Long axis on 3 MPR planes**					
Mean	52.0	0.882	0.876	0.3075	0.877
St. Deviation	17.70	0.063	0.035		0.059
Range	[31.75–86.61]	[0.719–0.935]	[0.8162–0.928]		[0.792–0.962]
**Long and short and few dropped points, on MPR**					
Mean	65.31	0.844	0.857	0.5205	0.825
St. Deviation	18.01	0.054	0.029		0.063
Range	[46.73–107.75]	[0.729–0.923]	[0.817–0.907]		[0.718–0.921]

**Figure 8:**
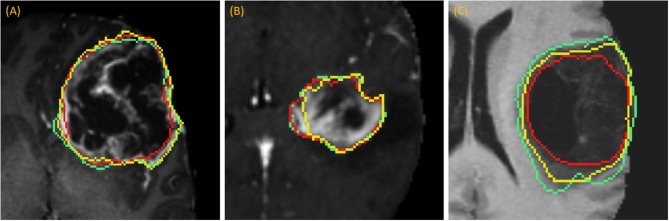
Accuracy Comparison: segmentation outlines by simulation (green), user (red) relative to the ground-truth (yellow) for one case in each of the three categories **(A)** drawing an axial long axis, **(B)** drawing long axes on all MPR planes, and **(C)** performing additional edits required by dropping points.

DSC scores obtained from manual segmentation and simulations are presented in Table 5. Simulated interaction performed marginally better in the first and third categories, while real interaction scored moderately better in the second category, though none of those differences were statistically significant. Further, DSC scores obtained by comparing user segmentation and simulation are in the same range as DSC scores with ground-truth. Given the three levels of user interaction, segmentation results are depicted in Figure 8. In most cases, the “between” DSC score was higher than both individual scores computed relative to ground-truth (Figure 8B). In Figure 8A, the “between” score is between the individual scores, which in this case, occurs because the user under-segmented the tumor area. Lastly, Figure 8C illustrates the third pattern of the “between” score lying below both individual scores. In this case the user under-segmented while the simulation over-segmented, resulting in great disparity between the two. Note, in the case depicted in Figure 8C there is no enhancing component surrounding the necrotic core, a typical presentation of a GBM, which would aid identification of tumor border and increase agreement between user and simulation.

## Discussion

As large-scale labeled image datasets are being curated for academic challenges and training DL models, the most common application tends to be the development of fully automatic methods for tumor segmentation that do not involve user interaction. One driver of this trend might be that developing and validating interactive algorithms becomes laborious as the datasets grow, owing to the time required to interact with every case in the validation dataset. Further, validation ideally occurs very frequently, interspersed between algorithm updates, and throughout the process of algorithm development. However, for a segmentation algorithm to be clinically applicable its outcome should be optimal, i.e., similar to the ground-truth, and therefore it's expected to require “some” user input (Langlotz et al., 2019). Our research aims to provide an approach to automate many aspects of user interaction and thus expedite large-scale validation.

Given a labeled dataset, it seems natural to employ it for validation by measuring the true longest axis, and then using that as an input to an interactive algorithm such as RPM. However, our results showed that the true longest axis would be a poor choice, as it scored the lowest of the four strategies listed in Table 2, where “sweeping” the long axis proved the optimal approach.

Further, our results showed a steady improvement in 3D accuracy as interaction increases on axial plane; DSC scores increased from 0.82 to 0.89. We demonstrate that drawing on MPR markedly improves accuracy vs. drawing on axial planes alone. Somewhat surprisingly, drawing a short axis in addition to the long axis made a rather insignificant improvement in DSC. Motivated by this finding, we changed RPM's design to accept multiple arbitrary lines rather than a single line constrained to be perpendicular to the long axis. Therefore, the user may draw the line through image content whose brightness needs to be sampled in order to complement the sampling already performed by the long axis.

Future work will be inspired by the results of Table 4, which suggest that accuracy in the 2D segmentations falls off moderately as the algorithm advances to the scout segmentations, and does so again during advancement to the 3D segmentation. Although each stage of the algorithm performs some machine learning to glean information from the results of the prior stage, perhaps more can be done in this regard. Future work will also investigate why additional editing operations worsened scores for certain patients as the statistical sampling from the initial long axis appears to have been better suited for application to 3D segmentation.

Table 5 reveals that accuracy was comparable between the simulated interaction and the real human interaction. Simulated interaction performed marginally better in the first and third categories, while real interaction scored moderately better in the second category. Human interaction scores lower when the segmenter and creators of ground-truth have a difference of opinion. This effect is somewhat canceled out by the fact that human interaction scores higher when the segmenter can apply more intelligence than that embodied by the simulation algorithm.

Timing results were extremely fast when comparing with the limited number of reports currently published; one study measured lung lesion contouring to require an average of 10.31 min (Velazquez et al., 2013). Our sub-minute timing confirms that RPM enables segmentation in routine clinical use.

For a technical description of how the algorithm compares with popular interactive methods, the reader is referred to Gering et al. (2018). Recent works have also introduced simulated user interaction to either identify the object and initiate segmentation (Xu et al., 2016) or refine the segmentation output obtained by DL (Wang et al., 2018; Zhou et al., 2019). In contrast, our goal in this manuscript was to simulate the approach a radiologist would follow for initiating a segmentation and providing input real time until the optimal result is achieved. When comparing the approach Xu et al. followed for object identification, their mode of interaction was to drop points, in contrast to our mixture of lines and points (Xu et al., 2016). Further, their objective was to generate tens of thousands of training samples and points are sampled randomly from the foreground and background object interiors with spacing constraints (Xu et al., 2016). For comparison with other methods which accept user strokes instead of just points, these randomly sampled points are expanded to circles of radius 5 pixels. Observe that human users would draw free-form strokes rather than perfect circles, so our method differs by its intention to more realistically mimic the actions of a human user, and by its purpose of facilitating continuous algorithm development. Last but not least, in our work we performed direct comparison of the outcome obtained from simulations with the outcome obtained by a human user to demonstrate that our simulations realistically capture real user interaction (Table 5 and Figure 8).

## Data Availability Statement

All datasets generated for this study are included in the article/supplementary material.

## Ethics Statement

Ethical review and approval was not required for the study on human participants in accordance with the local legislation and institutional requirements. Written informed consent for participation was not required for this study in accordance with the national legislation and the institutional requirements.

## Author Contributions

DG conceived the idea, developed the theory, and performed the computations. BY-M performed the user segmentations for the Timing Tests. DG contributed to the interpretation of the results. DG and AK wrote the manuscript. NM, AA, and TM provided critical feedback during manuscript preparation. LK, HK, JH, RC, and LP provided critical feedback in RPM discussions and design sessions. All authors approved the final manuscript.

### Conflict of Interest

DG, AA, and RC are named co-inventors on a patent application (Patent Application No. PCT/US2018/040473. DG, AA, JH, BY-M, LK, HK, RC, and LP are named co-inventors on a patent application (Patent Application No. PCT/US2019/059897). TM declares an equity interest and advisory role to HealthMyne, Inc. DG, BY-M, NM, AA, LK, HK, JH, RC, and LP declare equity interest to HealthMyne, Inc. AK declares salary support from The University of Texas MD Anderson Cancer Center, and HealthMyne, Inc.
